# Modern Sociology

**Published:** 1899-12-02

**Authors:** 


### Modern Sociology.

### CLINKER AND SLUDGE.

An important if not particularly attractive part of the work of sanitary authorities is the disposal of the sewage and other refuse of their districts. To the householder the whole matter ends when all refuse matter is removed from his domicile; but those in charge know that this is only the first step in sanitation. The old method of throwing sewage into rivers and ocean, to poison the harmless denizens thereof, if it does not, in one form or another, return to poison human beings, is going out of fashion. It is perceived that this does not solve the sewage problem, but only removes it a step farther off. Large towns have their sewage works, where the refuse of a great city can be turned into a pure effluent and an innocuous sludge. It is customary to offer visitors to these works a drink of the purified water that is recovered from the sewage. We do not know if the offer is very frequently accepted; probably prejudice is too firmly fixed in the human breast. But we are willing to believe that the liquid is pure and free from poisonous germs of any kind. There remains the solid matter—the sludge, as it is technically called.

In the opinion of sewage experts, the sewage question is the sludge question. The first idea was to get rid of the sludge. First water was tried, as has been said, then fire, which is still employed in several places; and, finally, the ever-obliging bacillus had been called in to help, and gorge his system with the waste products. Allowing that all these methods did the desired work, there still remained the further consideration of whether this was the best thing that could be done with the sludge. As one manager of sewage works recently said, all these methods were wasteful. They implied the destruction of substances, poisonous in their present condition, but capable of being turned to most useful account. The despised sludge is an admirable fertiliser. Was it not, then, the height of folly to go to great expense to destroy a substance for which the land was crying out, and in which in some other form was being imported in great quantity? But there was a serious prejudice against the use of sewage for manure. A few years ago farmers would hardly take the cakes of dried sludge as a gift. But this prejudice is now vanishing, and it is eagerly bought at a price of 1s. per ton. At this price it is probably a very cheap manure. At the Dalmarnock Sewage Works, where the sewage of Glasgow is treated, there was no demand for this sludge two years ago, and now the demand is so great that the manager has booked orders for eight thousand tons since June. Moreover, at present the Glasgow Sub-Committee on Sewage Disposal is considering an offer for a million tons, the contract to extend over ten years, and the price to be 1s. 6d. per ton. This offer comes from a syndicate who propose to establish works for the manufacture of chemical manures, of which the sludge will be the basis. There is, therefore, the expectation that what has hitherto been a cause of expense may hereafter be a source of revenue. Whether the demand for sludge will be sufficient to induce small towns to establish sewage works is not certain. The initial expense is serious, and could be justified only if there was a prospect of some part of it being recouped by the sale of the sludge.

So much for sewage. There remains the ordinary ash-pit refuse. Some part of this, also, might be turned into manure, but there remains much of apparently doubtful utility. Yet it has been found that all can be turned to account, and at a comparatively small expense. A refuse destructor is not a very expensive thing to set up, and it will dispose of rubbish in a speedy and sanitary manner. The product resulting from this destruction is known as "clinker," and amounts to about 30 per cent. of the mass consumed. Until 1894 this residuum was regarded as mere waste, to be got rid of in the easiest way possible. In the town of Bradford, we are told by the Cleansing Superintendent, they have 300 tons of ash-pit refuse to deal with every day. This is burned at four destructor stations. The cleansing department has thus 100 tons of clinker to get rid of every day. Until two years ago this was carted away for a distance of two miles, the carting costing the town £1,000. But in the neighbourhood there were four mortar mills, and it was found that these could make use of the clinker. Last year these mills bought from the Bradford Corporation 6,112 loads, at the price of 8d. a load, the total sum received from this source amounting to over £2,000. The town is thus, between what is saved and what is gained, £3,000 to the good. Among other uses to which the clinker can be put is the making of paving-slabs. Machinery for this has been erected at Bradford. It is estimated that granite-faced slabs can be put on the market, made from this clinker, at about two-thirds of the price of stone ones.

The economic disposal of town refuse of all sorts is only now beginning to be seriously thought of, but the results are sufficiently encouraging to lead us to think that the sanitary departments of our towns may yet become, at least to some extent, self-supporting. To get rid of refuse in a cleanly and sanitary fashion is a good thing, to make money out of the doing of it something even better.

